# Comparative transcriptomics analysis revealing flower trichome development during flower development in two *Lonicera japonica* Thunb. cultivars using RNA-seq

**DOI:** 10.1186/s12870-020-02546-6

**Published:** 2020-07-17

**Authors:** Jianjun Li, Chenglin Ye, Cuifang Chang

**Affiliations:** 1 Green Medicine Biotechnology Henan Engineering Laboratory, Engineering Technology Research Center of Nursing and Utilization of Genuine Chinese Crude Drugs in Henan Province, College of Life Science, Henan Normal University, Xinxiang, China; 2 State Key Laboratory Cell Differentiation and Regulation, College of Life Science, Henan Normal University, Xinxiang, Henan China

**Keywords:** *Lonicera japonica* Thunb., Trichome development, Yujin 1, Comparative transcriptomics, Flower

## Abstract

**Background:**

*Lonicera japonica* Thunb. (*L. japonica*) has the functions of clearing away heat and detoxifying, broad-spectrum antibacterial and anti-virus, etc. More than 70% of anti-inflammatory and cold Chinese patent medicines contain *L. japonica*. Trichomes comprise specialized multicellular structures that have the capacity to synthesize and secrete secondary metabolites and protect plants from biotic and abiotic stresses. The extraction of trichome secretions has great commercial value. However, little is known about the trichome formation mechanism in *L. japonica*. Therefore, the study of trichome development between different varieties provides a basis for selecting suitable planting resources.

**Results:**

Here, we present a genome-wide comparative transcriptome analysis between two *L. japonica* cultivars, toward the identification of biological processes and functional gene activities that occur during flowering stage trichome development. In this study, the density and average lengths of flower trichomes were at their highest during three-green periods (S2). Using the Illumina RNA-Seq method, we obtained 134,304 unigenes, 33,733 of which were differentially expressed. In an analysis of 40 differentially expressed unigenes (DEGs) involved in trichome development, 29 of these were transcription factors. The DEGs analysis of plant hormone signal transduction indicated that plant growth and development may be independent of gibberellin (GA) and cytokinine (CTK) signaling pathways, and plant stress may be independent of jasmonic acid (JA) and ethylene (ET) signaling pathways. We screened several genes involved in the floral biosynthesis of odors, tastes, colors, and plant hormones, and proposed biosynthetic pathways for sesquiterpenoid, triterpenoid, monoterpenoid, flavonoid, and plant hormones. Furthermore, 82 DEGs were assigned to cell cycles and 2616 were predicted as plant resistance genes (PRGs).

**Conclusions:**

This study provides a comprehensive characterization of the expression profiles of flower development during the seven developmental stages of *L. japonica*, thereby offering valuable insights into the molecular networks that underly flower development in *L. japonica*.

## Background

*Lonicera japonica* Thunb. (*L. japonica*) is an extensively used traditional Chinese medicine that may be employed in both food and medicine [1]. *L. japonica* is incorporated within approximately 1/3 of traditional Chinese medicine preparations, and is also widely used in health care products, cosmetics, foods, and more [1]. The dry buds or open flowers constitute the medicinal components of Chinese medicinal *L. japonica*, which are used for the prevention and treatment of severe acute respiratory syndromes, H1N1 influenza, and hand-foot-and-mouth disease [2]. Pharmacological studies have also demonstrated that *L. japonica* flowers possess antibacterial, anti-inflammatory, anticancer, anti-diabetic, anti-oxidative and antiviral properties, as well as various other pharmacological effects [3–6]. Consequently, *L. japonica* is referred to as a “plant antibiotic” [7]; its adaptability is very robust and it is widely distributed across the nation [7]. However, the yields and quality of *L. japonica* are highest in Fengqiu of Henan and Shandong Provinces. A new variety of *L. japonica* (Yujin 1) was selectively bred by our research group from the main cultivar of Fengqiu ‘Damaohua’, which possesses the attributes of large buds, high yields, strong resistance, and a high content of active elements (chlorogenic acid (CGA) and luteoloside) [8].

Plant trichomes are epidermal outgrowths that protect plants from the attack of herbivorous insects, which develop even when plants are grown under optimal conditions [9]. Interestingly, plasticity allows plants to respond to insect attacks by increasing the population and density of trichomes in new growing leaves, stems, and flowers [10]. Trichomes exhibit high morphological variations and can be divided into several classes, which can be unicellular or multicellular, glandular or glandless, as well as branched or unbranched. In many plant species, trichomes are glandular multicellular structures that are able to generate and store several valuable secondary metabolites such as terpenes, alkaloids, phenols, sterols, and aromatic oils, that are important resources not only for plant development and defense, but also for the support of human life and the treatment of disease [11].

The processes of initiation and development for trichomes involve a complex genetic network. Our knowledge about this developmental process is still limited, but genes controlling glandular trichome initiation and morphogenesis have recently been identified [12]. These genes may be segmented as activators and suppressors according to whether the epidermal cells can differentiate into trichomes. Activators include a multimeric complex, known as the trichome activator complex, which is formed by a *R2R3 MYB* protein GLABROUS1 (GL1), two redundant trichome formation bHLH proteins, GLABRA3 (GL3) and ENHANCER OF GLABRA3 (EGL3), a WD40 repeat containing protein, TRANSPARENT TESTA GLABRA 1 (TTG1), and the enhanced expression of TRIPTYCHON AND CAPRICE 2 (TC2) and GLABRA2 (GL2), which promote trichome differentiation [13, 14]. Suppressors include CAPRICE (CPC), TRIPTYCHON (TRY), ENHANCER OF TRIPTYCHON AND CAPRICE 1 (ETC1), ENHANCER OF TRIPTYCHON AND CAPRICE 2 (ETC2), ENHANCER OF TRIPTYCHON AND CAPRICE 3 (ETC3), and TRICHOMELESS 1 (TCL1), all of which are members of the *R3 MYB* transcription factor family, which can also form complexes with TRANSPARENT TESTA GLABRA 1 (TTG1) and GL3 to inhibit the differentiation of epidermal hair cells [15, 16]. In addition, the latest studies reported that HD-ZIP IV, bHLH95, DELLA, GL6 and Nck-Associated Protein 1 (NAP1) are involved in the trichome initiation [12, 17–20], and PARC6 is critical for plastid morphogenesis in trichome [21].

Cyclins are the best-known positive regulators of cell proliferation, and their molecular mechanisms in the cell-cycle transition are conserved in eukaryotes [22]. It has been reported that *SlCycB2* plays a critical role in reproductive organ development, multicellular trichome initiation [23], secondary metabolite biosynthesis, and defense [24]. The study shows that *Arabidopsis* nucleoporin CONSTITUTIVE EXPRESSION OF PR GENES 5 (CPR5) controls trichome cell death through the core cell cycle regulator CYCLIN-DEPENDENT KINASE INHIBITOR (CKI) [25]. Phytohormones serve critical roles in plant growth and development. Recent studies have shown that the biosynthetic and signal transduction pathways of gibberellin (GA), cytokinine (CTK), jasmonic acid (JA), and salicylic acid (SA) are involved in the initiation of trichome development [13]. However, phytohormones sometimes have antagonistic functions due to competition. Both GA and CTK stimulate trichome formation and floral induction; however, exogenous GA applications may inhibit the effects of CTK treatments, as GAs are able to block CTK signaling [26].

Trichome is closely related to plant resistance and the formation of volatile oils; thus, it is an important index for resource evaluation and species identification. However, there are no reports to date that elucidate the molecular mechanisms of trichome formation on *L. japonica* flowers. The quality of *L. japonica* primarily depends on the developmental period of the flower [27]. In addition, flowering-time genes affect the initiation of trichome [13]. Therefore, this study employed ‘Yujin 1’ and ‘Damaohua’ flowers at different developmental stages to study the molecular mechanisms of trichome formation via morphology, transcriptome, and bioinformatics, etc., so as to provide opportunities for improving the germplasm of *L. japonica* through genetic engineering technologies, while cultivating new varieties with high yields, high quality, multi-resistance, and high efficiency.

## Results

### Morphology, density and length of flower trichome

**Fig. 1:**
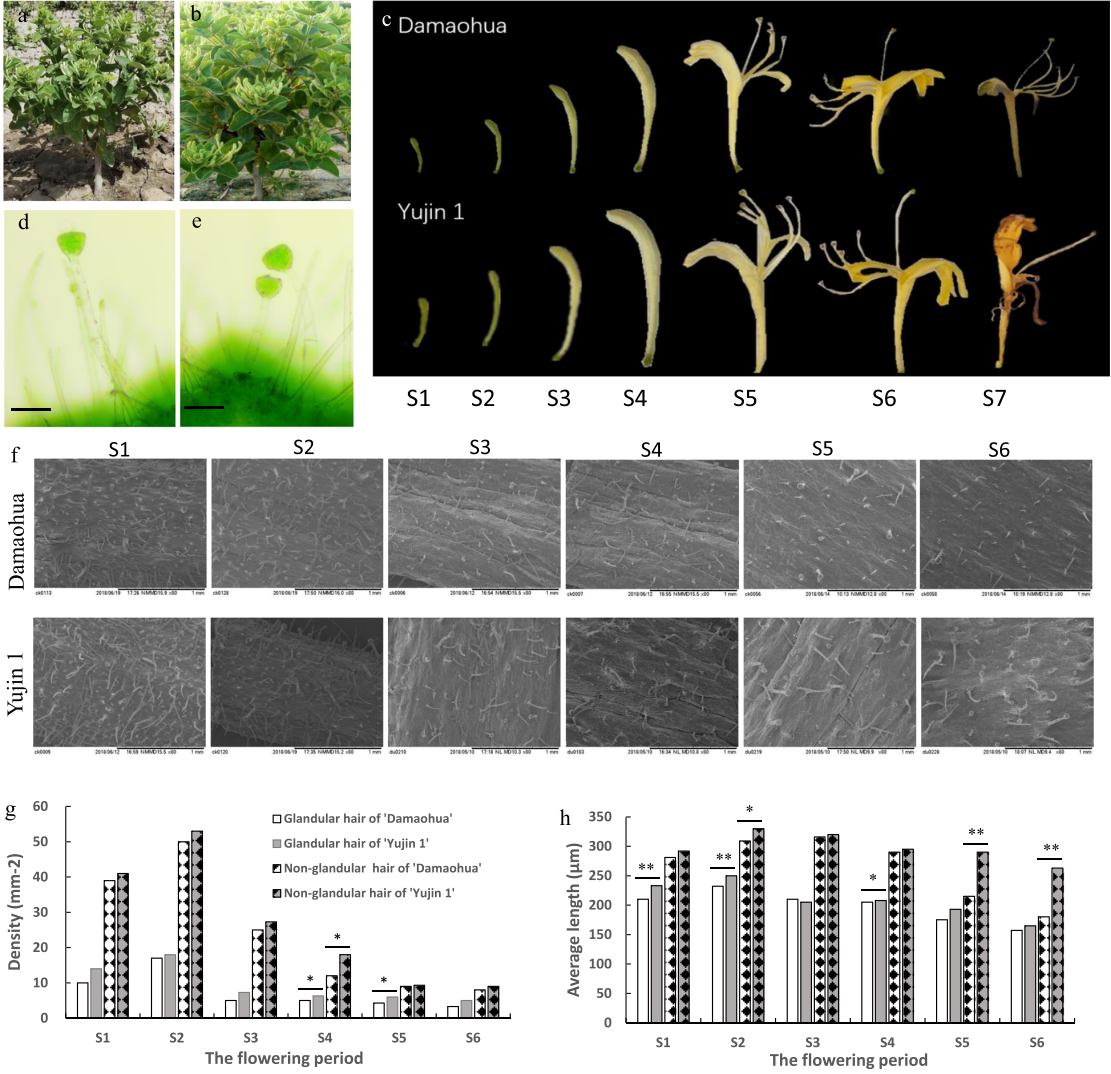
Morphology, density, and length of flower trichomes in ‘Damaohua’ and ‘Yujin 1’. **a** ‘Damaohua’ *L. japonica* plant. **b** ‘Yujin 1’ *L. japonica* plant. **c** The morphology of flowers in seven developmental stages of two *L. japonica* varieties.. **d** The morphology of flower trichome at the two white stages (S3) in ‘Damaohua’ (bar = 50 μm). **e** The morphology of flower trichome at the two white stages (S3) in ‘Yujin 1’ (bar = 50 μm). **f** The arrangement of trichomes on buds or flowers samples at six flowering stages of ‘Damaohua’ and ‘Yujin 1’ by SEM (bar = 1 mm). **g** Comparison of the density of glandular and non-glandular hairs in ‘Damaohua’ and ‘Yujin 1’. **h** Comparison of the lengths of glandular and non-glandular hairs in ‘Damaohua’ and ‘Yujin 1’. The annotations of the column color are the same as g’s. Data are presented as the mean ± standard error (SE), *n* = 5. *p < 0.05, **p < 0.01

A new variety of high-quality *L. japonica*, designated as ‘Yujin 1’, was identified and developed by our research group, which has the characteristics of large flower buds, as well as dense and long trichomes (Figs. 1a, b, c). The flower trichomes of both species of *L. japonica* were composed of glandular hair and non-glandular hair (Figs. 1d, e). The glandular hair is multicellular, and the head of the glandular hair contains pigment. The hair stalks of ‘Yujin 1’ are obviously longer, composed of 2 ~ 3 cells, and the cells are longer.

The non-glandular hairs of ‘Damaohua’ and ‘Yujin 1’ are single cell thick walls with long or short bristles, respectively. Consistent with these results, we further examined their morphologies and measured the density and length of trichomes on buds or flowers samples at six flowering stages of ‘Damaohua’ and ‘Yujin 1’ under a scanning electron microscope (Fig. 1f, Tables S1, S2). The results indicated that the density of glandular and non-glandular hairs was greatest at S2, and there was significant difference between ‘Yujin 1’ and ‘Damaohua’ at S4 (Fig. 1g). The lengths of non-glandular hairs were longest at S2, and the lengths of glandular hairs were significantly different between ‘Yujin 1’ and ‘Damaohua’ at S1 and S2. Further, the length of non-glandular hairs showed significant differences between ‘Yujin 1’ and ‘Damaohua’ at S5 and S6 (Fig. 1h).

### RNA-seq and de novo assembly

To compare comprehensive gene expression profiles and characterization of the two species *L. japonica* flowers at seven stages of development, transcriptome sequencing and analysis were performed. Following the removal of adaptor and low quality sequences, the clean reads were assembled into expressed sequence tag clusters (contigs) and de novo assembled into transcripts using the Trinity in paired-end method, which yielded a total of 134,304 unigenes with an average length of N50 of 1642 bp, and GC content of 45.75% (Tables S3, S4). Thus, the assembly quality of the transcriptome was satisfactory.

### Function annotation and classification

**Fig. 2:**
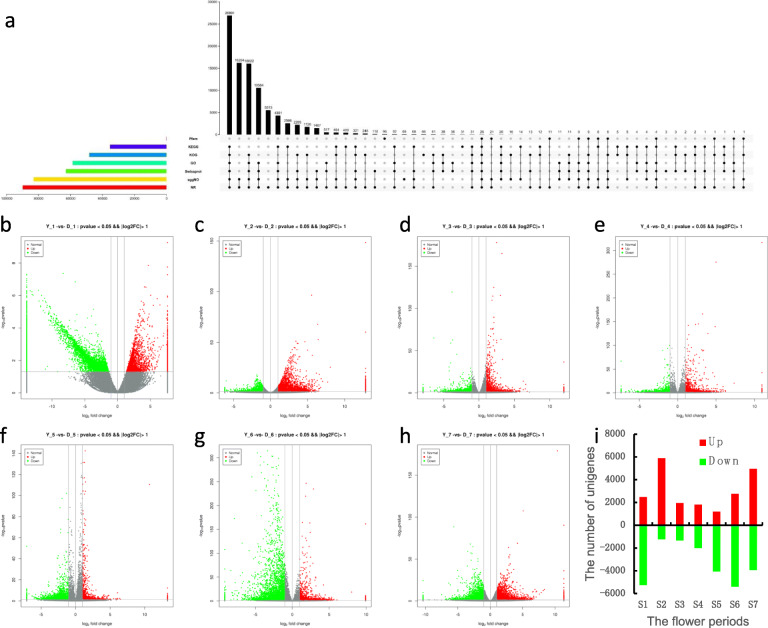
Annotation and DEGs of *L. japonica* transcriptome. **a** Venn diagrams for each database annotation of *L. japonica* transcriptome. **b-h** Volcano plot of upregulated and downregulated genes of *L. japonica* in the seven stages. **i** The number of upregulated and downregulated genes in the seven stages

The assembled unigenes were annotated by common databases including the NR, Swiss-Prot, KEGG, KOG, eggNOG, GO, and Pfam, to which approximately 66.94, 46.80, 26.27, 35.89, 61.82, 43.67, and 0.14% of unigenes were mapped, respectively (Table S5). Among the total unigenes, a total of 90,338 (67.26%) were annotated. The number of unigenes annotated by only one database were 5513, 3, 31, 66, 69, 0, 96 for NR, Swiss-Prot, KEGG, KOG, eggNOG, GO, and Pfam, individually (Fig. 2a).

### Comparative analysis of differentially expressed unigenes (DEGs) in seven stages

To acquire insights into functional and regulatory dynamics during flower development, pairwise differential analysis (‘Yujin 1’ vs ‘Damaohua’) was conducted on the expression levels of DEGs by using the RPKM method for the seven developmental stages, respectively. All references to the direction of differential expression refer to expression in the ‘Yujin 1’ cultivar relative to the ‘Damoahua’ cultivar. There were 7725, 7132, 3290, 3826, 5245, 8145 and 8907 DEGs at seven stage, respectively (Figs. 2b-h). Combining the DEGs for all flowering stages, we obtained a total of 33,733 DEGs. As shown in the figures, the number of DEGs at S1 and S2 was higher than that at other stages (Fig. 2i).

### GO and KEGG pathway analysis of DEGs

All of the transcriptome unigenes were used as background, and obviously enriched GO terms were obtained for the DEGs in the seven stages (Figs. S1A-G). For example, the response to chitin included in the biological process was enriched at S2, S3, S4, S5, S6, and S7; plant-type hypersensitive response was enriched at S1, S2, S3, S4, and S6; and defense response was enriched at S2, S3, S4, and S6. Apoplast, cell wall, integral component of membrane, and plasma membrane included in the cellular component were enriched at S1, S2, S3, S4, and S6. ADP binding included in the molecular function was enriched at S1, S2, S3, S4, and S6; DNA binding transcription factor activity was enriched at S2, S3, S4, and S6; and xyloglucan:xyloglucosyl transferase activity was enriched at S1, S2, S5, and S6.

In the KEGG pathway analysis, the prevailing pathways were as follows: phenylpropanoid biosynthesis and plant hormone signal transduction were enriched at all stages; starch and sucrose metabolism was enriched at S1, S2, S3, S4, S6, and S7; alpha-Linolenic acid metabolism was enriched at S1, S2, S3, S4, and S5 (Figs. S1H-N, Table S6).

### DEGs and TFs related to trichome development and validation by qRT-PCR

**Fig. 3:**
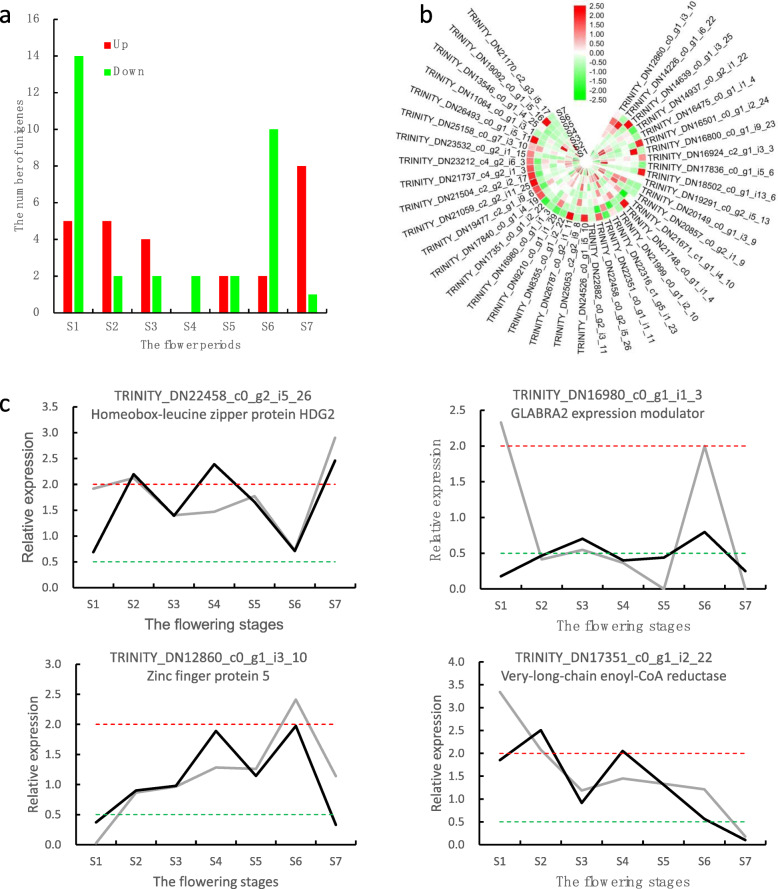
Identification and validation of potential unigenes associated with trichome development, and their expression levels at different stages of *L. japonica*. **a** Numbers upregulated or downregulated in the seven stages; **b** Transcript expression analysis for unigenes associated with trichome development. Changes expression levels are represented by color; green indicates a lower expression level and red indicates a higher expression level. **c** Real-time qPCR validation of four genes involved in trichome development. The black line indicates qPCR results, grey line indicates RNA-Seq results, whereas the red and green dotted lines indicate differentially expressed up and down levels

To explore the molecular basis of the differences in trichome development between ‘Yujin 1’ and ‘Damaohua’, we identified important functional genes involved in *L. japonica* trichome development. The sequencing results revealed that 160 unigenes were related with trichome development, and 40 (mapped into 19 genes) were significantly upregulated or downregulated in the seven stages (Figs. 3a, b). Among these, 11 unigenes, including *TRY*, *SUPPRESSOR OF K+ TRANSPORT GROWTH DEFECT1 (SKD1*), and *BLISTER* were involved in trichome branching (GO:0010091), eight unigenes, including *FAS1*, *GL2*, and *GDPDL3* were involved in trichome differentiation (GO:0010026), 17 unigenes, including *GTL1*, *SPIRRIG*, and *FPP4* were involved in trichome morphogenesis (GO:0010090). Further, *ECR* was involved in trichome papilla formation (GO:1905499); *RAC1* was involved in seed trichome differentiation (GO:0090379); and *RNF115_126* was involved in trichome elongation (GO:0090378). Of the 40 trichome development-related unigenes, 29 were transcription factors, which belonged to 12 transcription factor families, including bHLH, bZIP, C2H2, and so on (Table S7).

To verify the sequencing data, we selected four unigenes related to trichome development for qRT-PCR verification. The primers were designed using Primer Primer 5.0 (Table S8). Generally, the expression patterns determined by real-time qPCR were consistent with those obtained by RNA-Seq (Fig. 3c), which confirmed the accuracy of the RNA-Seq results reported in this study.

### DEGs related to signal transduction

**Fig. 4:**
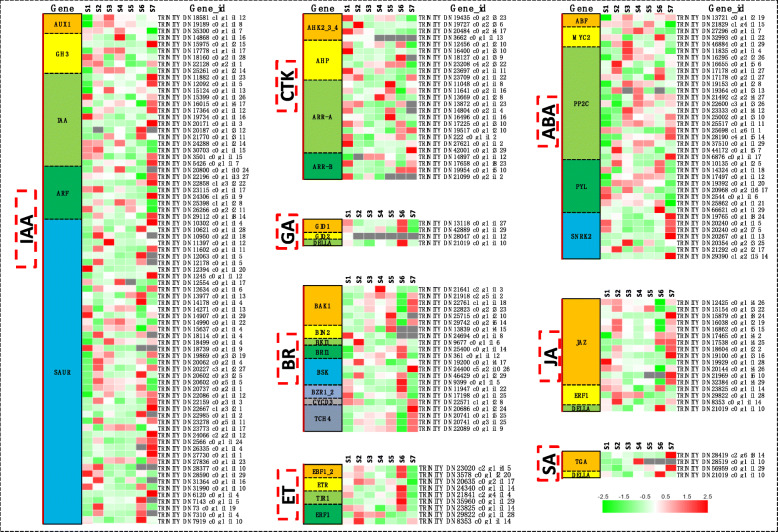
Expression profiles of plant hormone signal transduction related genes in the seven flowering stages of *L. japonica*

In the analysis of the KEGG pathway, the plant hormone signal transduction, phosphatidylinositol signaling system, Wnt signaling pathway, adenosine monophosphate activated protein kinase (AMPK) signaling pathway, phospholipase D signaling pathway, and transforming growth factor beta (TGFβ) signaling pathway were enriched (Fig. S2; Table S9). Among these, plant hormone signal transduction played a critical role in trichome development. Our transcriptome data and cluster analysis revealed that multiple metabolism-related genes associated with plant hormones were enriched and corresponding signaling pathways are also activated (Fig. 4). There were 77, 25, 4, and 22 DEGs in trichome development that were involved in plant growth- and development-related hormones, including auxin (indoleacetic acid, IAA), CTK, GA, and brassinosteroid (BR), respectively. Additionally, there were 37, 9, 17, and 4 DEGs in trichome development that were associated with stress-related hormones, including abscisic acid (ABA), ethylene (ET), JA, and SA, respectively.

### DEGs related to secondary metabolites

Secondary metabolites play major roles in the adaptation of plants to the environment and in overcoming stress conditions, while also contributing to the specific odors, tastes, and colors of plants. Glandular hairs can secrete chemicals to protect against biological and abiotic stresses and signal transduction, including organic acids, polysaccharides, proteins, polyphenols, alkaloids, and terpenoids. The analysis of pathways enriched by KEGG revealed that phenylpropanoid biosynthesis related to plant disease resistance was enriched at S1-S7 stages. The biosynthesis of sesquiterpenoid, triterpenoid, and monoterpenoid related to odors and tastes were enriched at S1, S2, S3, and S6. The biosynthesis of flavonoids related to colors was enriched at S1. The biosynthesis of tryptophan metabolism, glycerolipid metabolism, terpenoid backbone, and brassinosteroid, related to plant growth- and development-related hormones were enriched at different stages. The biosynthesis of carotenoid, cysteine and methionine metabolism, unsaturated fatty acids and phenylalanine, tyrosine and tryptophan were also enriched at different stages (Table 1, Table S10).

**Table 1:** Statistically enriched secondary metabolites pathways identified using KEGG in differentially expressed transcripts during flower development of *L. japonica*

KEGG pathway ID	Pathway definition	ListHits	PopHits	%	The flower stages
ko00940	Phenylpropanoid biosynthesis	164	396	41.41%	S1, S2, S3, S4, S5, S6, S7
ko00909	Sesquiterpenoid and triterpenoid biosynthesis	39	82	47.56%	S1, S2, S3, S6
ko00941	Flavonoid biosynthesis	17	65	26.15%	S1
ko00902	Monoterpenoid biosynthesis	16	32	50.00%	S2
ko00400	Phenylalanine, tyrosine and tryptophan biosynthesis	57	264	21.59%	S2
ko00380	Tryptophan metabolism	67	308	21.75%	S7
ko00561	Glycerolipid metabolism	101	332	30.42%	S1, S2, S6
ko00900	Terpenoid backbone biosynthesis	65	296	21.96%	S3
ko00906	Carotenoid biosynthesis	50	129	38.76%	S1, S2
ko00270	Cysteine and methionine metabolism	99	520	19.04%	S5
ko00625	Chloroalkane and chloroalkene degradation	49	128	38.28%	S2, S3, S4, S7
ko00100	Steroid biosynthesis	84	239	35.15%	S1, S2, S4, S5
ko00140	Steroid hormone biosynthesis	7	29	24.14%	S5
ko00905	Brassinosteroid biosynthesis	9	21	42.86%	S1
ko00592	alpha-Linolenic acid metabolism	59	153	38.56%	S1, S2, S3, S4, S5
ko00591	Linoleic acid metabolism	31	56	55.36%	S1, S3, S4, S6
ko01040	Biosynthesis of unsaturated fatty acids	47	215	21.86%	S3, S5
ko00400	Phenylalanine, tyrosine and tryptophan biosynthesis	57	264	21.59%	S2

ListHits: the number of differentially expressed genes in the KEGG term; PopHits: the number of all genes annotated to the term

### Cell cycle related DEGs

**Fig. 5:**
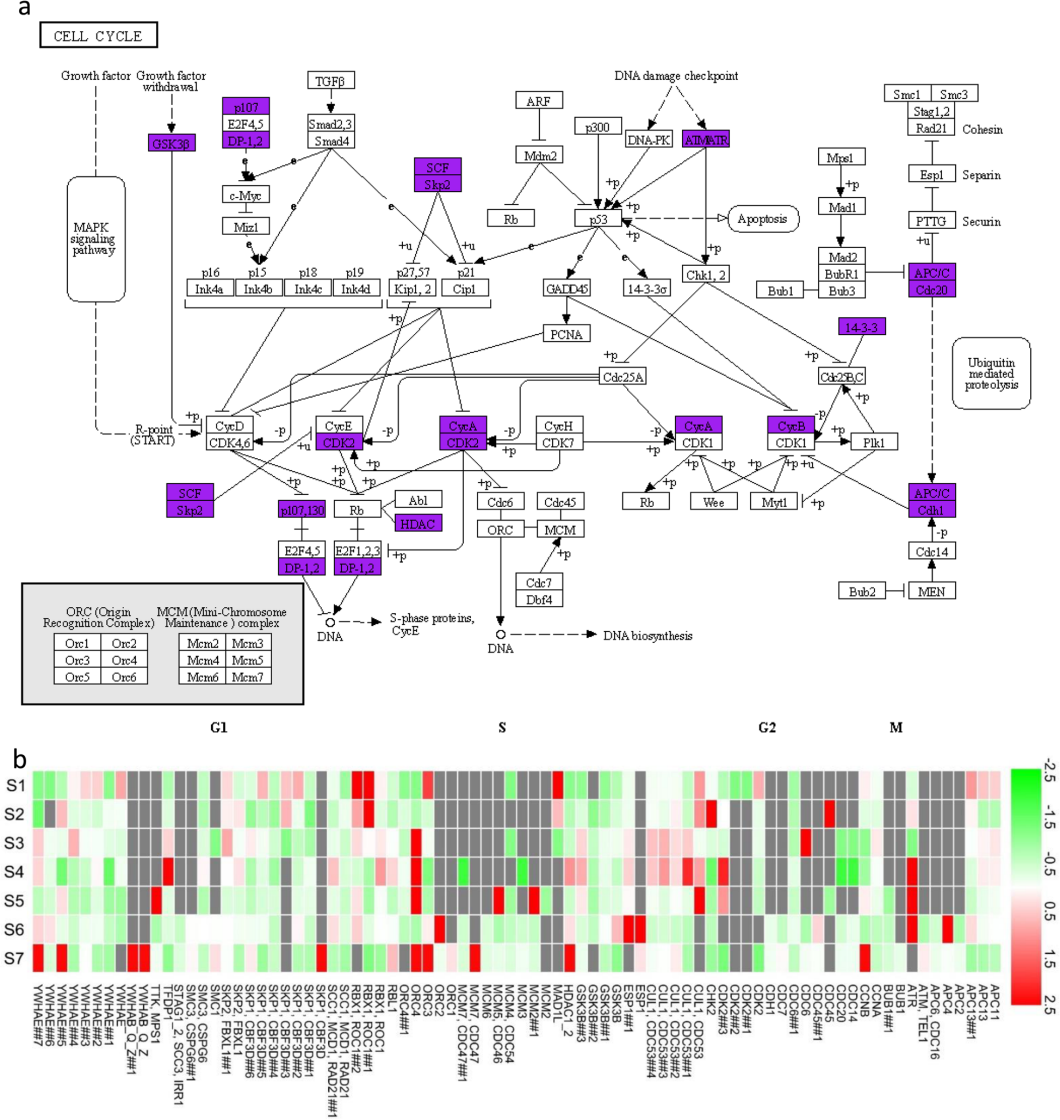
Unigenes involved in the cell cycle and their expression profiles in the seven flowering stages of *L. japonica*. **a** Cell cycle map of KEGG. Purple genes indicate differentially expressed unigenes. **b** Transcript expression analysis for unigenes associated with cell cycle. Changes in expression level are represented by color; green indicates a lower expression level and red indicates a higher expression level

Trichome development is closely related to the cell cycle. KEGG analysis revealed that 82 unigenes were involved in the cell cycle, which could map into 45 genes (Fig. 5a). Among these, *APC11*, *CDC7*, *CDC53*, *CDC54*, and *CDC47* were up-regulated in S1, whereas *CDK2* was down-regulated; *CDC7*, *CDC45*, *GSK3B*, *YWHAE*, and *MCM2* were up-regulated in S2, and no gene was down-regulated; *MCM6* and *CHK2* were up-regulated in S3; *CHK2*, *CDK2*, *BUB1*, and *ORC1* were up-regulated in S4; No gene was upregulated in S5; *RBX1*, *CCNA*, *SKP1*, and *MCM2* were up-regulated in S6, with the important thing being that *CDK2* expression was down-regulated; and 35 genes, including *CCNB*, were up-regulated in S7, with only *BUB1*, *TFDP1*, and *GSK3B* being down-regulated (Fig. 5b).

### DEGs related to plant resistance

**Fig. 6:**
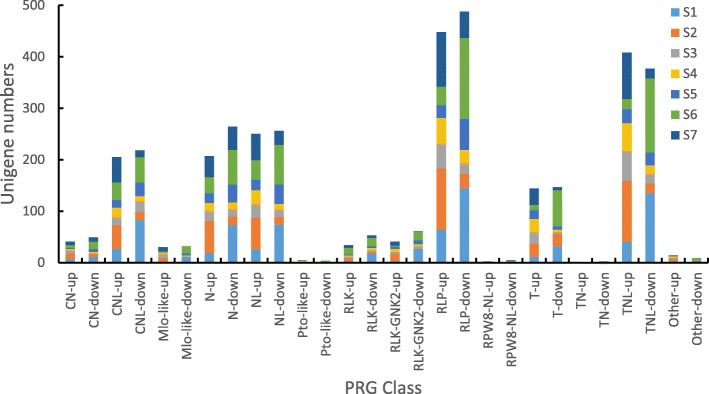
The distribution of PRG Class in the seven flowering stages of *L. japonica*. PRGs are divided into 15 functional categories, and definitions can be found in the http://prgdb.crg.eu/wiki/ Category:Classes

Glandular trichomes play an essential role in the protection of plants against biotic and abiotic stresses. We compared the expression of plant resistance genes (PRGs) at different flowering stages of ‘Yujin 1’ vs ‘Damaohua’, and found that a total of 2616 DEGs annotated to the PRG database and distributed across 13 classes, including CN, CNL, Mlo-like, N, NL, Pto-like, RLK, RLK-GNK2, RLP, RPW8-NL, T, TN, TNL, and unknown (Table S11). Among these, the distribution of PRGs in the RLP class was the highest, with the TNL class being second; However, the proportion of up-regulated PRGs was highest in the TNL class (Fig. 6).

## Discussion

The *Lonicera japonica* Thunb*.* flower is a perennial, evergreen, twining vine that has double-tongued flowers that open white and fade to yellow, which have been employed for the treatment of various diseases for many years, with its potential effects described in numerous studies [3]. Trichomes are epidermal protuberances that protect plants from herbivorous insect attack, which develop even when the plants are grown under optimal conditions [28]. Trichomes are also present on the flowers of *L. japonica*. In many plant species trichomes are glandular multicellular structures that are able to produce, distribute, and store toxic substances for protection against insect attack [29]; however *L. japonica* trichomes are unicellular and non-glandular structures (Fig. 1). The new variety of high-quality *L. japonica* called ‘Yujin 1’, was identified and created by our research group, which has the characteristics of larger flower buds, as well as denser and longer trichomes than ‘Damaohua’.

Although the DEGs analyses of nine tissues and transcriptional regulation during flower development in *L. japonica* were performed using RNA-Seq [27, 30], the availability of data on developmental trichome expression profiles and comparisons of flower transcriptomes at different stages remains generally limited. To reveal the molecular mechanisms of trichome development of two species of *L. japonica* flowers at seven stages, transcriptome sequencing and analysis were performed. Using the Illumina RNA-Seq method, we obtained 134,304 unigenes, of which 90,338 (67.26%) covered the entire life cycle of the plant. Based on these transcriptomic data, we obtained 33,733 candidate genes that were differentially expressed at seven flowering stages.

At present, extensive research on the formation and development of trichomes in *Arabidopsis thaliana*. They found that the formation and development of trichomes are regulated by GA and CTK signaling, and transcription factors including MYB, bHLH, C2H2 [13]. Sequencing results revealed that 40 DEGs (mapped into 19 genes) were significantly upregulated or downregulated in the seven stages; 29 of these were transcription factors. The regulation of trichome development was affected by GA and CTK hormones through the transcriptional regulation of the GIS clade genes: *GIS*, *GIS2*, and *ZFP8* [13]. The trichome activator GL2 was activated by GIS2 and ZFP8, while GIS positively regulated some of the members of the trichome activation complex-GL1, TTG1m, and GL3/EGL3, which in turn triggered GL2, and simultaneously, the R3-MYB repressor genes. The R3-MYB members that included CPC, ETC1, ETC2, ETC3, TCL1, TCL2, and TRY served as trichome initiation repressors [13, 31]. GTL1 was present within the nucleus during the post-branching trichome development stages, where its loss of function leads to an increase in nuclear DNA content; however, only in those trichomes that completed branching [32]. ECR, also known as eceriferum 10 (CER10), was found to represent a new allele of *glh6*, which promoted trichome papillae development [33]. It was reported that a microRNA knock-down of the ABIL3 gene led to a distorted trichome phenotype [34]. In this study, most of the unigenes of the GA and CTK signaling pathway were upregulated at S1, while the expressions of *GL2*, *GTL1*, and *TRY* was downregulated at S1. However, *ABIL3* and *ECR* were significantly increased at S1, which may have caused the trichome of ‘Yujin 1’ to be denser and longer.

A key and unique feature of glandular trichomes, relative to their size, is the capacity to synthesize and secrete large quantities of a limited number of metabolites: primarily terpenoids, but also phenylpropanoids and flavonoids [11]. In this study, we screened several genes associated with the floral biosynthesis of odors, tastes, colors, and plant hormones, and proposed a biosynthetic pathway for sesquiterpenoid, triterpenoid, monoterpenoid, flavonoid, and plant hormones biosynthesis at different flower stages. AtSKD1 contributes to vacuolar protein trafficking and subsequently, to the maintenance of the large central vacuoles of plant cells in early stages of trichome development [35]. The expressions of *SKD1* were upregulated at S2 and S3, which may have been correlated with the secretion of metabolites in glandular trichomes.

Trichome development is intimately related to the cell cycle [36]. It was reported that SlCycB2 plays a critical role in reproductive organ development, multicellular trichome initiation, and secondary metabolite biosynthesis [24]. In this study, CCNB did not change from S1–6, while *APC11*, *CDC7*, *CDC47*, *CDC53*, and *CDC54* were up-regulated at S1, and CDK2 was down-regulated. It was reported that the ANAPHASE PROMOTING COMPLEX/CYCLOSOME (APC/C) (APC11) is critical for cell cycle synchronization in the endosperm of *Arabidopsis thaliana* [37]. The upregulated expression of *APC11* may be closely related to the density of ‘Yujin 1’. The roles of *CDC7*, *CDC47*, *CDC53*, and *CDC54* in plants are rarely reported, which warrant further study.

Plant trichomes frequently function as the first line of defense against biotic and abiotic stresses via space hindrance [38]. In this study, we compared the expression of plant resistance genes (PRGs) at different flowering stages of ‘Yujin 1’ vs ‘Damaohua’, which found that a total of 2616 PRGs. Simultaneously, the stress-related jasmonic acid signaling pathway and ethylene signaling pathway were also enriched at S1 and S2, which may have been closely related to the increased resistance of ‘Yujin 1’.

## Conclusions

This work presents a genome-wide comparative transcriptome analysis firstly between two *L. japonica* cultivars, toward the identification of biological processes and functional gene activities on trichome development that occur during flowering stage. This study provides a comprehensive characterization of the expression profiles of flower development during the seven developmental stages of *L. japonica*, and screened several genes may involve in the floral biosynthesis of odors, tastes, colors, and plant hormones, and proposed biosynthetic pathways for sesquiterpenoid, triterpenoid, monoterpenoid, flavonoid, and plant hormones. Furthermore, 82 DEGs were assigned to cell cycles and 2616 were predicted as plant resistance genes. In all, this study provides a theoretical basis for the identification of *Lonicera japonica* Thunb. varieties and the selection of new varieties, and lays a solid theoretical foundation for the further study of the molecular mechanism of its specific characteristics. It has important academic and application value. In the subsequent work, genetic transformation and phenotypic identification of the selected genes will be conducted to further study their effect on trichome development.

## Methods

### Plant materials

The two breeds of *L. japonica* were five-year ‘Damaohua’ and ‘Yujin 1’ that grow in the resource garden of the College of Life Sciences, Henan Normal University (N35°18 ‘13.71″, E113°55′ 15.05). ‘Damaohua’ and ‘Yujin 1′ were identified as honeysuckle by Li at Henan Normal University, China. The fresh flower buds or flowers of ‘Damaohua’ and ‘Yujin 1′ were collected separately from five plants at seven stages: (S1) young bud stage; (S2) three-green stage; (S3) two-white stage; (S4) great-white stage; (S5) silver stage; (S6) golden stage; (S7) fade stage. During sample collection, the flowers of five plants were combined and regarded as one biological replicate that representing each stage, and three independent replicates were performed. Partial flower materials were flash frozen in liquid nitrogen following collection and stored at − 80 °C.

### Microscopic and scanning electron microscope (SEM) observation of trichome

The fresh buds or flowers of two varieties at S3 were sliced and added to distilled water on microscope slides for observation and photography. In addition, we used a scanning electron microscope (SEM) (TM3030Plus, Hitachi, Japan) to observe the arrangement of trichomes on *L. japonica* bud or flower samples. According to methods outlined by Ning et al. [39], the bud or flower samples (3 mm × 3 mm) were soaked three times in a phosphate buffer solution (pH 7.3) for 1 min, respectively. The samples were then transferred into 2.5% glutaraldehyde solution at 4 °C for > 24 h, and then dehydrated using a series of ethanol (30, 50, 70, 80, 90, 95, 100 and 100%) mixtures at 4 °C for 30 min, respectively, followed by a series of tert-butyl alcohol (70, 80, 90, 100%) mixtures at room temperature for 20 min to remove the ethanol. After being dried in a freeze-drying box (VFD21S) at 4 °C for 30 min, the samples were sprayed with a 12.5–15 nm gold layer and examined/photographed from multiple different perspectives using a scanning electron microscope. This process was repeated three times for each sample, and five sites were selected for imagery acquisition, and the density and length of glandular hairs and non-glandular hairs were measured.

### RNA isolation and library construction

The total RNA was extracted using a mirVana miRNA Isolation Kit (Ambion) following the manufacturer’s protocol [40]. RNA integrity was evaluated using an Agilent 2100 Bioanalyzer (Agilent Technologies, Santa Clara, CA, USA). The samples with an RNA Integrity Number (RIN) ≥ 7 were subjected to further analysis. The mRNA of each sample was isolated from the total RNA by using beads with oligo (dT), and were added with a fragmentation buffer to cleave the mRNA into short fragments, which were then employed as templates for the synthesis of first-strand cDNA using random hexamer primers. These libraries were developed using TruSeq Stranded mRNA LTSample Prep Kit (Illumina, San Diego, CA, USA) according to the manufacturer’s instructions.

### Sequencing, de novo assembly and annotation

The libraries above were sequenced using an Illumina HiSeq X Ten sequencer (Illumina Inc., USA) and 150 bp paired-end reads were generated. The preparation and sequencing of the cDNA library were performed at Shanghai OE Biotech. Co., Ltd., Shanghai, China. Raw data (rawreads) were processed using Trimmomatic [41]. Reads containing ploy-N and low quality reads were removed to obtain clean reads. Following the removal of adaptor and low quality sequences, the clean reads were assembled into expressed sequence tag clusters (contigs) and de novo assembled into transcripts using Trinity [42] (vesion: trinityrnaseq_r20131110) in paired-end method. The longest transcript was selected as a unigene based on the sequence similarity and length for subsequent analysis.

The functions of the unigenes were annotated as NCBI non-redundant protein (NR), Clusters of orthologous groups for eukaryotic complete genomes (KOG), Gene Ontology (GO), Swiss-Prot, evolutionary genealogy of genes: Non-supervised Orthologous Groups (eggNOG), and Kyoto Encyclopedia of Genes and Genomes (KEGG) databases using diamond software, and mapped to Pfam databases by HMMER. The search was conducted using Blastx [43] with a threshold E-value cut-off of 10^− 5^.

### Unigene quantification, analysis of differentially expressed unigenes (DEGs), cluster analysis, GO and KEGG enrichment

The FPKM [44] and read count values of each unigene were calculated using bowtie2 [45] and eXpress [46]. The DEGs were identified using the DESeq [47] functions estimate Size Factors and nbinom Test. The *P* value < 0.05 and foldchange > 2 or foldchange < 0.5 was set as the threshold for significant differential expression. Hierarchical DEGs cluster analysis was performed to explore transcript expression patterns. DEGs GO enrichment and KEGG pathway enrichment analyses, respectively, were performed using R, based on hypergeometric distribution.

### Identification and expression analysis of transcription factors (TFs)

PlantTFDB (http://planttfdb.cbi.pku.edu.cn/index.php) is a plant transcription factor database that includes the sequences of 58 plant transcription factor families from 165 plant species [48]. The sequence of unigenes were Blastx aligned to the transcription factor database, and the best of these, with E value of less than 1e-5, was screened as the annotation information of the unigene. Candidates that contained DNA binding domains were recognized by GO annotation for the final TF identification. Differentially expressed TFs (DETFs) between samples were identified using the value of Fragments Per Kilobase of transcript Per Million fragments mapped (FPKM) with |log2(fold change)| > 1, *p* value ≤0.05 and *q* value ≤0.05 [49].

### Identification and expression analysis of plant resistance genes (PRGs)

The Plant Resistance genes database (http://prgdb.crg.eu/wiki/Main_Page) contains more than 112 resistance genes and 104,335 candidate Resistance Genes [50]. According to the specific domains, the PRGs are divided into 15 category (does not contain Unknown) functions, and their definitions can be found in the http://prgdb.crg.eu/wiki/Category:Classes. The sequence of unigenes were Blastx aligned to the PRG database, and the best of these with an E value of less than 10^− 5^ was screened as the annotation information of the unigene. The Expression analysis of the PRGs were the same as the TFs.

### qRT-PCR

The identical RNA samples as the RNA-seq experiments were used for qRT-PCR. The yield of RNA was determined using a NanoDrop 2000 spectrophotometer (Thermo Scientific, USA), and the integrity was evaluated using agarose gel electrophoresis stained with ethidium bromide. Quantification was performed using a two-step reaction process: reverse transcription (RT) and PCR. Quantification was performed using the 2^−ΔΔCT^ method, and data were normalized to the ACT2/7 transcript [51].

### Statistical analysis

Significant differences were calculated using a one-way ANOVA analysis with a Turkey test and a significance level at *p* ≤ 0.05 and *p* ≤ 0.01 using SPSS 19.0 software. All expression analyses were performed in three replicates. The reported values represented arithmetic averages of three replicates, and the data was expressed as a mean plus or minus standard deviation (mean ± SD).

## Supplementary information

**Additional file 1: Figure S1.** Summary of top 10 GO terms and top 20 KEGG pathway assignments for the *L. japonica* flower transcriptome. A-G: The top 10 GO terms in S1, S2, S3, S4, S5, S6, and S7. H-N: The top 20 KEGG pathways in S1, S2, S3, S4, S5, S6, and S7.

**Additional file 2: Figure S2.** Transcript expression analysis for unigenes associated with signal transduction in different stages of *L. japonica*. A: Plant hormone signal transduction; B: phosphatidylinositol signaling system; C: Wnt signaling pathway; D: AMPK signaling pathway; E. phospholipase D signaling pathway; F: TGF-beta signaling pathway. Changes in expression levels are represented by color; blue indicates a lower expression level and red indicates a higher expression level.

**Additional file 3: Table S1.** Comparison of the density of glandular and non-glandular hairs in ‘Damaohua’ and ‘Yujin 1’ (mm-2). **Table S2.** Comparison of the lengths of glandular and non-glandular hairs in ‘Damaohua’ and ‘Yujin 1’ (μm). **Table S3.** Assembly summary of transcriptomic data. **Table S4.** Preprocessing results of sequencing data quality. **Table S5.** Annotation results of the unigenes. **Table S6.** Summary of top 10 GO terms and top 20 KEGG pathway assignments for the *L. japonica* flower transcriptome. **Table S7.** DEGs involved in trichome development. **Table S8.** Primers used for analysis of gene expression by qRT-PCR. **Table S9.** DEGs involved in signal transduction. **Table S10.** DEGs involved in secondary metabolites. **Table S11.** DEGs involved in plant resistance.

## Data Availability

The datasets used and analyzed in the current study are available from the corresponding author on reasonable request. Sequences have been deposited in NCBI Sequence Read Archive under project PRJNA637952 (https://www.ncbi.nlm.nih.gov/bioproject/PRJNA637952).

## Abbreviations

ABA: Abscisic acid
BR: Brassinosteroid
CGA: Chlorogenic acid
CTK: Cytokinine
DEGs: Differentially expressed unigenes
ET: Ethylene
GA: Gibberellin
IAA: Indoleacetic
JA: Jasmonic acid
PRGs: Plant resistance genes
SA: Salicylic acid
