# Supplementary figures and images for: Comparative transcriptomics analysis revealing flower trichome development during flower development in two Lonicera japonica Thunb. cultivars using RNA-seq
Source: BMC Plant Biol. 2020 Jul 17;20:341. doi: 10.1186/s12870-020-02546-6 (PMC7368687; doi:10.1186/s12870-020-02546-6)

## Slide 1
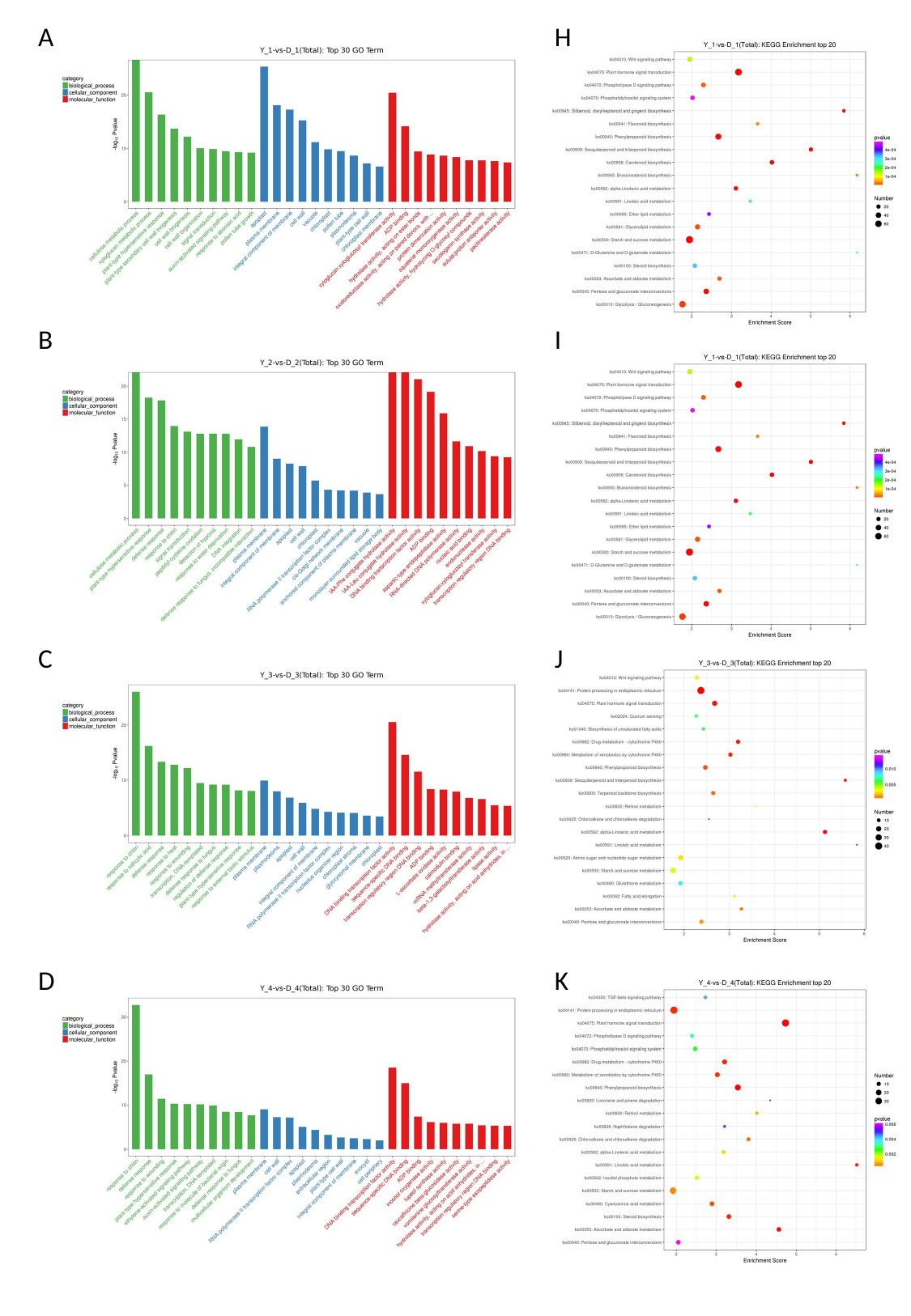

A
H
B
I
C
J
D
K

## Slide 2
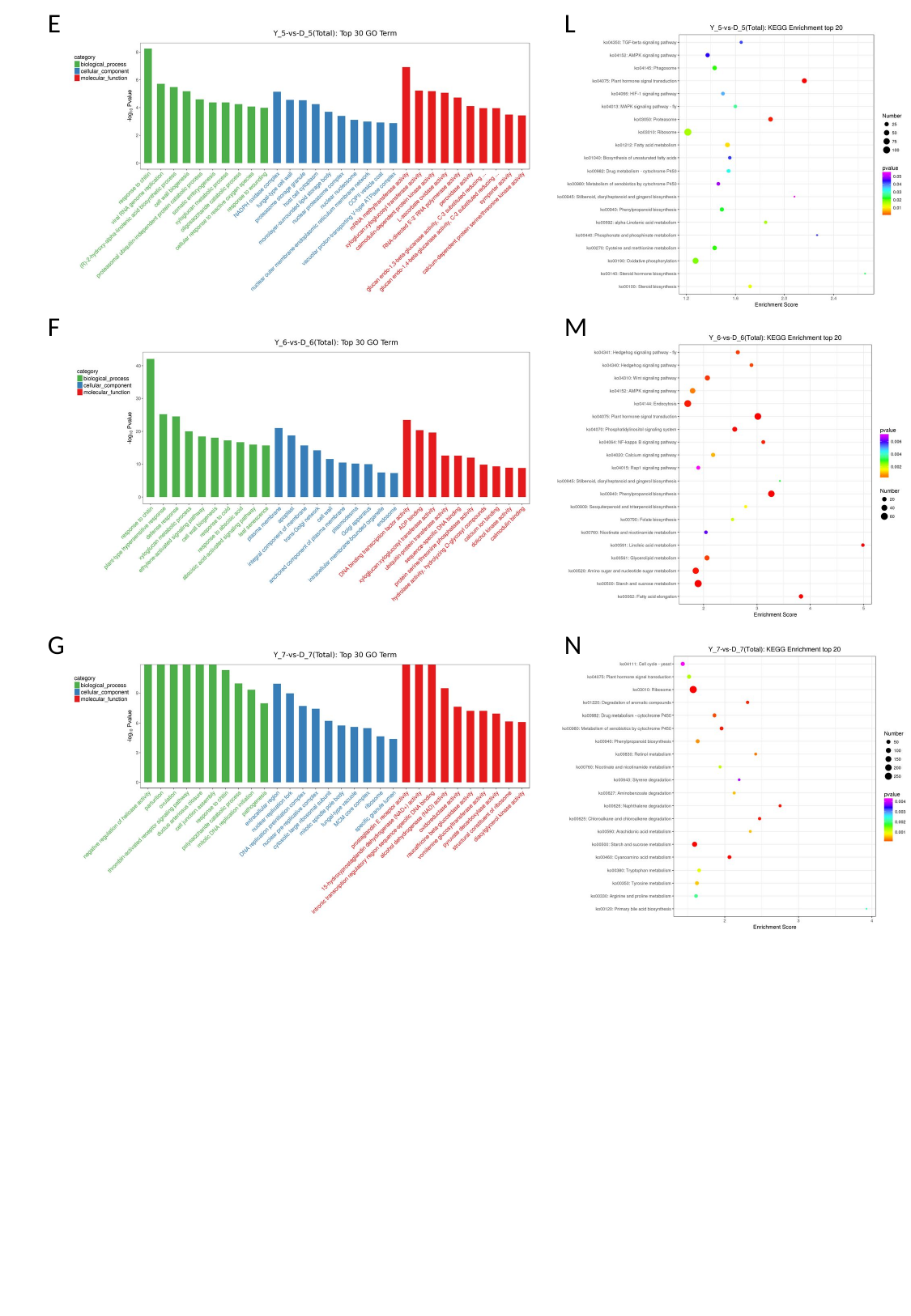

E
L
F
M
G
N

Supplement: Supplementary file 1 — Additional file 1: Figure S1. Summary of top 10 GO terms and top 20 KEGG pathway assignments for the L. japonica flower transcriptome. A-G: The top 10 GO terms in S1, S2, S3, S4, S5, S6, and S7. H-N: The top 20 KEGG pathways in S1, S2, S3, S4, S5, S6, and S7. [file 12870_2020_2546_MOESM1_ESM.pptx]

## Slide 1
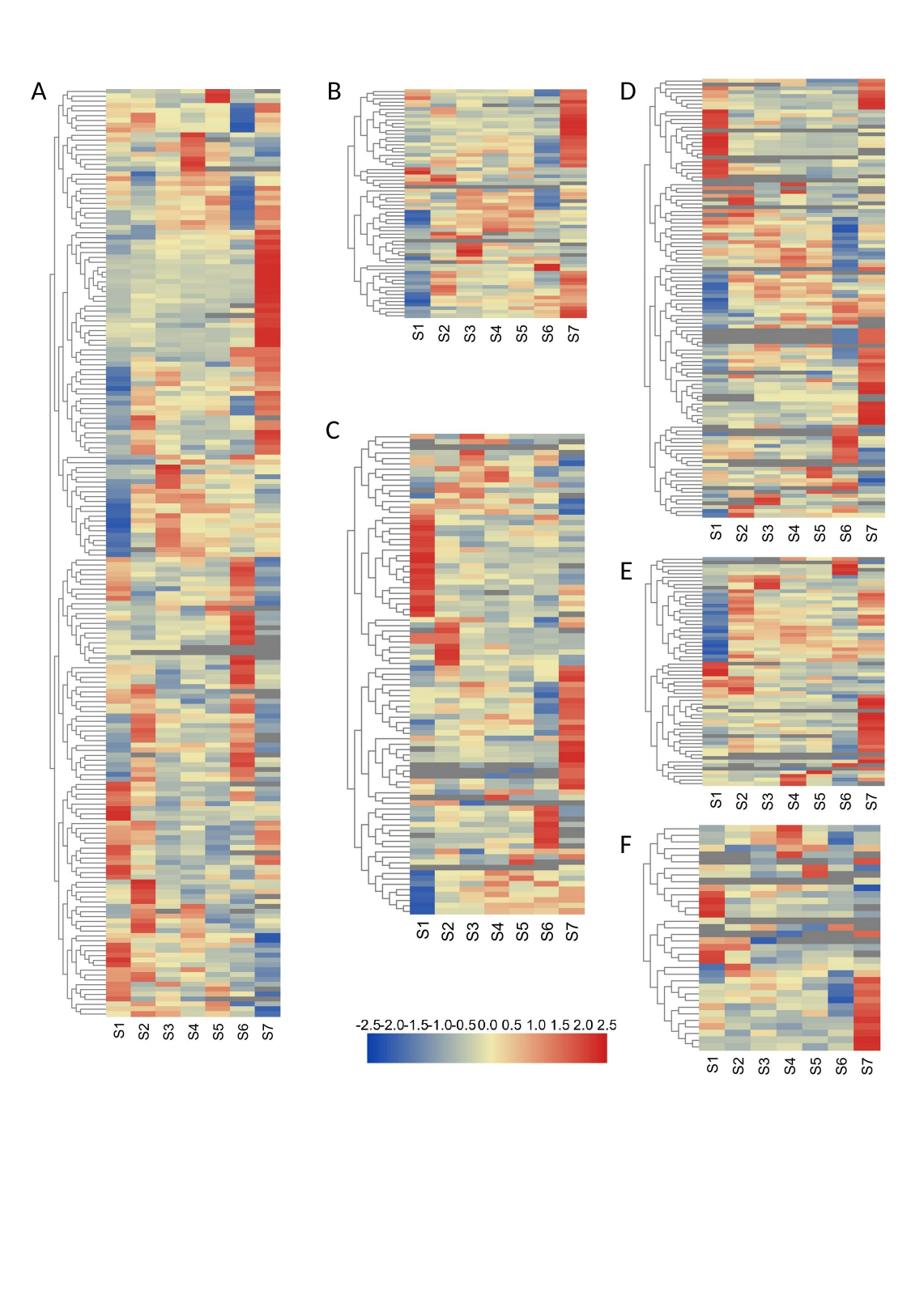

B
D
A
C
E
F

Supplement: Supplementary file 2 — Additional file 2: Figure S2. Transcript expression analysis for unigenes associated with signal transduction in different stages of L. japonica. A: Plant hormone signal transduction; B: phosphatidylinositol signaling system; C: Wnt signaling pathway; D: AMPK signaling pathway; E. phospholipase D signaling pathway; F: TGF-beta signaling pathway. Changes in expression levels are represented by color; blue indicates a lower expression level and red indicates a higher expression level. [file 12870_2020_2546_MOESM2_ESM.pptx]
